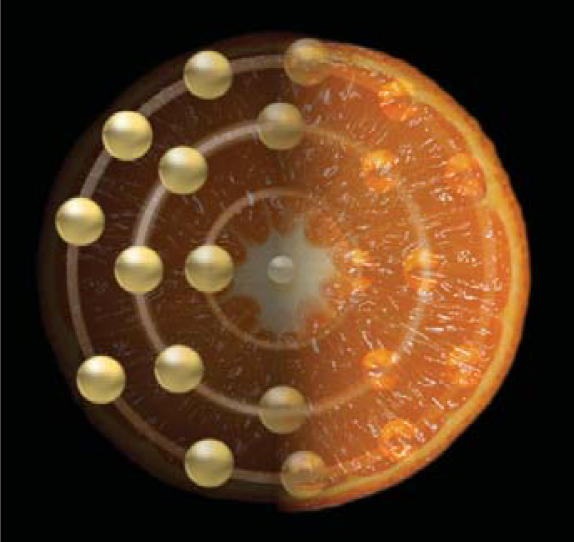# Chemical Exposures: Mutagenic Mix

**Published:** 2007-07

**Authors:** M. Nathaniel Mead

The carcinogenicity of hexavalent chromium (Cr[VI]) likely arises from its ability to cause DNA damage and mutations. Nonetheless, its mutagenicity has been found to be modest and typically detected only at high exposure concentrations. But recent research indicates that the mutagenic and other genotoxic abilities of Cr(VI) become amplified 10- to 20-fold in the presence of ascorbate, or vitamin C.

OSHA and IARC estimate that nearly 400,000 U.S. workers and several million more worldwide are exposed to Cr(VI) in the workplace, where chromium compounds (or chromates) serve as anticorrosion agents in protective coatings, as pigments in paints and plastics, and in chrome plating on tools, aircraft engine components, railroad wheel bearings, and automobile parts. Long recognized as a potent respiratory carcinogen, skin irritant, and kidney and liver toxicant, Cr(VI) was also shown in a May 2007 technical report by the National Toxicology Program to cause cancer in rodents exposed through drinking water.

Most cells grown in culture contain little or no detectable vitamin C, and the strikingly high genotoxic potential of Cr(VI) was revealed only when cultured human cells were supplemented with physiologically normal levels of ascorbate. “We found that doubling of ascorbate concentrations in human lung cells caused a very strong increase in the number of chromosomal breaks even with low doses of [Cr(VI)],” says principal investigator Anatoly Zhitkovich, an associate professor of medical science at Brown University.

The dosage level was one-quarter of the current federal standard for total Cr in drinking water of 100 ppb (Zhitkovich explains that the typical Cr content in contaminated water is predominantly Cr[VI] because Cr[III] is poorly soluble). “Overall,” he says, “we consistently found that the potentiating effects of vitamin C on genetic damage were always much stronger with low doses of chromium.” Zhitkovich and his colleagues believe that their findings, published in the January 2007 (number 2) issue of *Nucleic Acids Research*, can be relevant to low-level exposures in environmental settings.

Previous studies over the past decade have shown that vitamin C is a key reducer of Cr(VI) in major organs. Extracellular vitamin C helps detoxify Cr(VI) by reducing it to the less toxic form of Cr(III), which is thought to be unable to enter cells. But even normal levels of vitamin C inside the cell could be hazardous, and the new study sheds light on the specific ways in which the genotoxic potential of Cr(VI) is enhanced by ascorbate. First, the reduction of Cr(VI) by intracellular vitamin C results in dramatic increases in the formation of chromosomal breaks and gene mutations. Second, the increased genetic injury results from abnormal processing of Cr(VI)-induced damage by the DNA mismatch repair machinery of cells in the G_2_ phase of the cell cycle.

“This is the first truly thorough study on the role of ascorbate as an intracellular activator of chromate genotoxicity,” says Kent Sugden, an associate professor of chemistry at the University of Montana. “These findings beg to be followed up with whole animal studies that should be able to show the impact of vitamin C supplementation with regard to chromate metabolism and carcinogenicity.” The new findings, he adds, should serve as the basis for epidemiological studies of the impact of vitamin C on chromate metabolism in relation to cancer risk.

Sugden’s views are echoed by Max Costa, a professor and chairman in the Department of Environmental Medicine at the New York University School of Medicine. “People differ in their levels of ascorbic acid, but at least we can control those levels since . . . all of our ascorbic acid comes from our diet,” he says. “We now need a good population study that examines levels of ascorbic acid and chromate in red blood cells along with any associated genetic damage.”

Zhitkovich and his research team are now concentrating their efforts on figuring out how vitamin C increases genetic damage caused by Cr(VI). “Our current hypothesis is that vitamin C promotes the formation of bulkier forms of Cr[VI]–DNA damage that are more mutagenic and toxic to human cells,” he says.

## Figures and Tables

**Figure:**